# Intuitive Choices Lead to Intensified Positive Emotions: An Overlooked Reason for “Intuition Bias”?

**DOI:** 10.3389/fpsyg.2017.01942

**Published:** 2017-11-07

**Authors:** Geir Kirkebøen, Gro H. H. Nordbye

**Affiliations:** Department of Psychology, University of Oslo, Oslo, Norway

**Keywords:** decisions, intuition, biases, emotional consequences, personal involvement, responsibility

## Abstract

People have, for many well-documented reasons, a tendency to overemphasize their intuitions and to follow them, even when they should not. This “intuition bias” leads to several kinds of specific intuitive biases in judgments and decision making. Previous studies have shown that characteristics of the decision process have a tendency to “leak” into the experience of the choice outcome. We explore whether intuitive choices influence the experience of the choice outcomes differently from “non-intuitive,” analytic choices. Since intuition is feeling based, we examine in particular if intuitive choices have stronger affective consequences than non-intuitive ones. Participants in two scenario studies (*N* = 90; *N* = 126) rated the feelings of decision makers who experienced a conflict between two options, one intuitively appealing and another that appeared preferable on analytic grounds. Choosing the intuitive alternative was anticipated to lead to somewhat more regret after negative outcomes and, in particular, much more satisfaction with positive outcomes. In two autobiographical studies, one with psychology students (*N* = 88) and the other with experienced engineers (*N* = 99), participants were asked to provide examples of choice conflicts between an intuitive and a non-intuitive option from their own private or professional lives. Both groups showed a tendency to report stronger emotions, in particular positive, after intuitive choices. One well-established explanation for intuition bias focuses on the nature of people’s anticipated negative counterfactual thoughts if their decisions were to turn out badly. The present data indicate that intuitive choices intensify positive emotions, anticipated and real, after successful outcomes much more than negative emotions after failures. Positive outcomes are also more commonly expected than negative ones, when we make choices. We argue that markedly amplified emotions, mediated by stronger personal involvement, in the positive outcomes of intuitive versus non-intuitive choices, is an overlooked reason for intuition bias.

## Introduction

In daily life as well as in professional contexts, it is often possible to distinguish between decisions based on purposeful deliberation and reflection and more spontaneous, “intuitive” decisions. On a theoretical level, this distinction is reflected in various “dual process” theories (e.g., Chaiken and Trope, 1999; Evans, 2003), as encapsulated by, for example, the contrast between (fast) intuitive “System 1” and (slow) analytic “System 2” thinking (e.g., Stanovich and West, 2000; Kahneman, 2011). While it has been argued that these alternative ways of thinking should not be conceptualized as two separate “systems” (Keren and Schul, 2009), they do nonetheless reflect different approaches to a choice task. Researchers have long debated which approach is best under what conditions (e.g., Hammond et al., 1987; Wilson and Schooler, 1991; Gladwell, 2005; Hogarth, 2005, 2010; Dane et al., 2012). On the other hand, Inbar et al. (2010) found that, generally, decision makers tend to hold similar opinions about whether, and when, it is better to follow analytic procedures and when they should rather go with their gut.

In the present paper, we focus on choice dilemmas (similar to those studied by Simmons and Nelson, 2006) in which one option is (subjectively) more intuitively appealing, while the other, “non-intuitive” option is more strongly supported by the objective information at hand. Consider a hiring situation in which one candidate gives a better “impression” during the job interview and another candidate has a better record of education and relevant work experience. It is not obvious which candidate will end up being selected. Defined as an “intuitive choice” and a “non-intuitive choice,” this scenario represents a choice between the intuitive and the non-intuitive options, respectively, in such choice situations.

The purpose of the present paper is to go one step beyond the decision itself and examine whether the outcome of an intuitive choice will be evaluated differently from the same outcome of a non-intuitive choice. Such a difference may, in turn, predispose people to favor intuitive choices, and thus contribute to “intuition bias” in judgment and decision making.

### Intuition Bias

Psychological research has uncovered a widespread and strong tendency for people to rely on their intuitions and to follow them, even when they should not. This exaggerated tendency can be described as a general “intuition bias,” which lies at the root of a variety of psychological phenomena and leads to several more specific intuitive biases of judgment and decision making (e.g., Kahneman, 2011).

Many very different reasons for intuition bias have been posited. According to the two-system perspective, for instance, intuitive System 1 approaches often prevail over System 2 reasoning because people are unmotivated (cognitively lazy) or unable (cognitively overloaded) to apply the latter. However, even when neither laziness nor busyness are in play, people’s tendency to overemphasize their intuitions can emerge from intrinsic insufficiencies in System 2 corrections of System 1 intuitions—for example, insufficient adjustment after (intuitive) “anchoring” (e.g., Epley and Gilovich, 2006).

Another consideration regarding intuition bias with respect to decision making is the nature of people’s anticipated counterfactual thoughts concerning prospective outcomes of the decision if it were to turn out badly. An intuitive preference is automatic and unbidden. It is often experienced as something that is “given,” and to choose otherwise can be seen as an act of hubris that is likely to be punished by an undesirable result (e.g., Risen and Gilovich, 2008).

Prior studies have also suggested metacognitive explanations for a person’s strong tendency to go with their gut (e.g., Simmons and Nelson, 2006; Alter et al., 2007). For example, Simmons and Nelson (2006) demonstrated convincingly that people often choose intuitive alternatives rather than equally valid non-intuitive ones “because intuitions often spring to mind with subjective ease, and the subjective ease leads people to hold their intuitions with high confidence” (p. 409).

Thus, many plausible and well-founded explanations have been advanced with regard to intuition bias. In this article, we suggest still another reason, overlooked as far as we know, behind decision makers’ strong tendency to go with their gut.

### The Decision Process and How Outcomes Are Assessed

It is broadly accepted that behavior in general is shaped by consequences, and several studies have further found that characteristics of the decision process have a tendency to “leak” into the experience of the outcome (Keys and Schwartz, 2007). For example, Kirkebøen et al. (2013) established that decision reversals come with a cost: even if the final outcomes were equally good, those who ended up with a particular outcome after changing their mind reported markedly stronger post-outcome regret than those who achieved the same outcome without changing their mind.

In line with this, we expect that immediate intuitive and (more) reflective non-intuitive choices will influence differently the way outcomes are assessed, which, in turn, may contribute to intuition bias in decision making. So, what is the difference between how a decision maker experiences an outcome following an intuitive versus a non-intuitive choice?

### The Fluency of Action Selection and the Experience of Self-agency

The experience of “self-agency” has been described as “the feeling that one causes one’s own actions and their outcomes” (Aarts et al., 2009, p. 967). According to the traditional view, such a sensation is the result of a retrospective inference based on matching actual effects of an action with its expected effects (e.g., Moore and Haggard, 2008). However, Chambon and Haggard (2012) have demonstrated that the strength of experienced self-agency also reflects the fluency of action selection. Participants in their study were offered a choice of several alternatives, and the researchers influenced their selections through subliminal priming. It was found that, “when people responded to a target that was compatible with a preceding subliminal prime, they felt stronger sense of control over subsequent effect than when the preceding prime was incompatible” (Chambon and Haggard, 2012, p. 441). In short, they showed that a “sense of agency is partly generated prospectively, in advance of knowing the actual outcome of actions” (Chambon and Haggard, 2012, p. 441).

### Research Hypothesis

Intuition is feelings-based, and, as it is reasoned that characteristics of the decision process have a tendency to “leak” into the experience of the outcome, this implies that the outcome of feelings-based intuitive decisions will produce a stronger affective impact than will non-intuitive decisions.

We know that intuitions, or the intuitive alternative, usually come with subjective ease (e.g., Simmons and Nelson, 2006). In Chambon and Haggard’s (2012) terminology, this means that there should be a “greater fluency of action selection” when a person chooses the intuitive action versus the non-intuitive alternative. Based on Chambon and Haggard’s (2012) findings, we further expect that choosing the intuitive alternative will increase the decision maker’s experience of self-agency and thus the feeling of personal involvement, compared to what choosing the non-intuitive alternative would do. Stronger personal involvement should also make people more emotionally involved in their intuitive than in their non-intuitive choices.

Hence, we hypothesize that people are more personally involved and experience more intensified emotions, anticipated and actual, in response to the outcomes of intuitive versus non-intuitive choices. We will additionally argue that these intensified feelings reinforce people’s tendency to go with their gut when making decisions.

### Studies

We conducted two kinds of studies to explore our hypothesis: scenario studies and retrospective, autobiographical studies. In the scenario studies (Study 1 and Study 2), we presented the participants with choice situations within which the decision maker had two options, one supported by objective information (the non-intuitive choice) and the other supported by (holistic) intuitions—a gut feeling, an interview impression, and so on (the intuitive choice). In the retrospective studies (Study 3 and Study 4), we asked participants to report decision situations from their own life in which they had experienced a conflict between two such options. Participants in Study 3 were young psychology students, describing mostly choices they had made in their private life, whereas participants in Study 4 were experienced engineers who were asked to report professional decisions they had made.

## Study 1

### Method

The participants were undergraduate psychology students at the University of Oslo (*N* = 90; male = 22, female = 68; *M*_age_ = 23.44 years, *SD* = 5.31) who volunteered to take part in the study, which was conducted using pen and paper in a classroom setting.

The students were first asked about their preferences for intuitive versus non-intuitive choice alternatives, formulated in general terms: “Imagine you have a choice between two alternatives where your gut feeling favors one (the intuitive option) and rational analysis, facts, etc. favor the other (the non-intuitive option).” Participants were then presented with three concrete choice scenarios, as follows:

(1) *Employment of data manager*—a leader hires a data manager according to a job interview impression (intuitive option) or according to factual information about the applicants (non-intuitive option);
(2) *Choice of university*—a student chooses a university for a Master’s program according to the impression she got during a short visit to the universities (intuitive option) or according to factual information about the universities (non-intuitive option);
(3) *Choice of lodger*—selecting a lodger for a shared apartment based on the gut feeling after an interview (intuitive option) or based on references from previous landlords (non-intuitive option).

There were four versions (“V1” to “V4”) of each choice scenario, as follows:

(1) V1—trust intuition, negative outcome (i.e., the participant was asked to imagine that the protagonist had chosen the intuitively appealing alternative, which however turned out to be a failure);
(2) V2—do not trust intuition, negative outcome;
(3) V3—trust intuition, positive outcome;
(4) V4—do not trust intuition, positive outcome.

Each participant was presented with the general and the three concrete choice scenarios, with one scenario in version V1, another in version V2, etc. The four choice versions were randomly combined with the four choice situations.

For each choice situation, participants were asked (before the outcome was disclosed) to what extent they thought that the decision should be based on analysis (of facts) versus intuition, rated on a 9-point Likert-type scale (from 1 “*Analysis only*” to 9 “*Intuition only*”). Then, they were asked which option—the intuitive or the non-intuitive alternative—they themselves would have chosen. After receiving information about the outcome, they were asked to indicate how much they thought they would regret choices leading to negative outcomes (versions V1 and V2), and how satisfied they imagined they would be with choices having positive outcomes (versions V3 and V4).

We have posited that people are more personally involved following intuitive choices. If so, they should experience stronger feeling of responsibility for the outcomes of such choices. We therefore also asked, for all the scenarios, how much responsibility (guilt, credit) the participants expected to feel with respect to each outcome. All questions were answered according to 9-point Likert-type scales.

### Results

To the first question about how much they favored analysis and intuition in general, participants gave mean ratings on the 1–9 scale that indicated they would emphasize analysis and intuition almost equally (*M* = 4.96, *SD* = 1.60). Similar numbers of participants said that, in general, they would prefer the intuitive option (48.9%) and the non-intuitive alternative (51.1%). However, all three concrete scenario ratings were above the midpoint of the analysis–intuition scale, indicating a preference for relying on intuition: *Employment of data manager, M* = 5.77, *SD* = 1.87; *Choice of university, M* = 5.38, *SD* = 1.96; and *Choice of lodger, M* = 5.40, *SD* = 2.03). Correspondingly, a clear majority answered that they would themselves have chosen the intuitive alternative: respectively, *Employment of data manager*, 74.4% [a chi-square goodness-of-fit test indicates χ^2^ (1, *n* = 90) = 21.511, *p* < 0.001]; *Choice of university*, 56.3% [χ^2^ (1, *n* = 87) = 1.391, *p* = 0.238]; and *Choice of lodger*, 60.7% [χ^2^ (1, *n* = 89) = 4.056, *p* = 0.044]. Altogether, the intuitive options were chosen almost twice as often as the non-intuitive ones.

Overall, when we analyzed the three concrete scenarios combined, we found a significant tendency toward choosing the intuitive option (63.9 %, *p* < 0.001, 2-tailed, binomial test). The participants expected to be more satisfied when obtaining a positive outcome from an intuitive choice (*M* = 8.11, *SD* = 1.34) than from a non-intuitive choice (*M* = 7.39, *SD* = 1.28), *t*(129) = 3.100, *p* = 0.002, Cohen’s *d* = 0.55. They also expected to regret somewhat more obtaining a negative outcome from an intuitive choice (*M* = 7.19, *SD* = 1.52) than from a non-intuitive choice (*M* = 6.67, *SD* = 2.01); however, this difference failed to reach significance with a two-tailed test, *t*(135) = 1.716, *p* = 0.088, Cohen’s *d* = 0.29.

Similarly, the participants anticipated feeling more responsibility (credit) when ending up with a positive outcome from an intuitive choice (*M* = 7.03, *SD* = 1.55) versus a non-intuitive choice (*M* = 6.40, *SD* = 1.77), *t*(128) = 2.158, *p* = 0.033, Cohen’s *d* = 0.38. They also expected to feel more responsibility (guilt) when ending up with a negative outcome from an intuitive choice (*M* = 7.34, *SD* = 1.42) versus a non-intuitive choice (*M* = 6.84, *SD* = 1.93), but this difference failed to reach significance, *t*(135) = 1.717, *p* = 0.088, Cohen’s *d* = 0.30.

### Discussion

When participants were asked about choices in general, they reported that they did not expect to feel more strongly about the outcomes of intuitive versus non-intuitive choices. However, in the concrete scenarios, they anticipated stronger feelings following the outcomes of intuitive choices.

Previous studies have focused on how decision makers frame their feelings/thoughts in the case of *negative* outcomes of intuitive choices versus deliberate choices (e.g., Kruger et al., 2005; Risen and Gilovich, 2008). In contrast, the present study found a significant difference only between projected emotions in *positive* outcomes of intuitive versus non-intuitive choices, whereas the difference between the anticipated feelings in negative outcomes were not found to be significant.

It was found that participants felt more responsibility (credit, guilt) for the outcome of intuitive choices, especially in the case of positive outcomes. This result may indicate that the decision makers consider themselves more personally involved in their intuitive choices (e.g., Woolfolk et al., 2006; see also the section “General Discussion” below).

In sum, the findings of Study 1 support our hypothesis that people are more personally involved in their intuitive choices and experience more intensified emotions in response to the outcomes of intuitive versus non-intuitive choices, in particular in relation to positive decision outcomes.

## Study 2

### Method

The participants in Study 2 were undergraduate psychology students at the University of Oslo (*N* = 126; male = 22, female = 104; *M*_age_ = 25.60 years, *SD* = 5.92), who volunteered to take part in the study. In Study 1, the participants were asked to anticipate their *own* feelings when choosing either intuitively or non-intuitively, in a between-subjects design. In Study 2, we asked participants to anticipate *other* decision makers’ feelings, in a combination of between- and within-subjects designs. The study was conducted using pen and paper in a classroom setting.

As in Study 1, participants were presented with different choice situations in which the decision maker had two options, one favoring intuitive feelings and the other reasons. Two scenarios were used: the “*Employment of data manager*” scenario from Study 1 and an “*Ordering of goods for a fashion chain*” scenario in which a shop manager orders clothing items according to her own intuitions (intuitive option) or according to an analysis of market trends, etc. (non-intuitive option).

**FIGURE 1:**
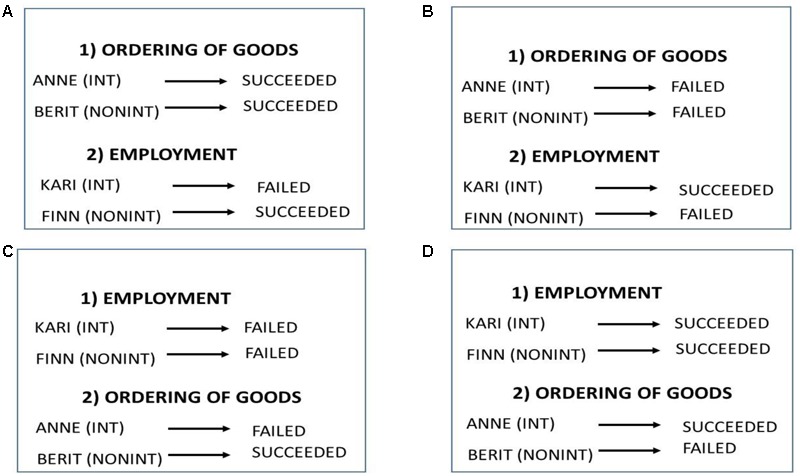
The choices in the four groups (conditions): **(A), (B), (C)** and **(D)**, in Study 2.

The participants were randomly assigned to one of four groups (conditions): (A), (B), (C), or (D). In all the groups, they were presented with one choice from each of the two scenarios. The order of the choice scenarios varied (see **Figure 1**). The first was “Ordering of goods” for participants in groups (A) and (B), and “Employment” for participants in groups (C) and (D). For each choice, participants were asked to compare two hypothetical decision makers, one of whom followed her intuition whereas the other selected the alternative incorporating more objective information. In one of the choice scenarios, both the intuitive and the non-intuitive decision maker succeeded or failed. In the other scenario, one of them failed and the other succeeded.

For instance, in group (A), the “Ordering of goods” scenario described two sales managers, Anne and Berit, who ordered the spring collection of clothes according to an intuitive or a non-intuitive approach, respectively, and they were both successful. Group (A) participants were questioned as follows:

• ANNE did not take the analyses of previous sales into account. She followed her intuition, and succeeded. How satisfied (on a scale of 1 to 7) do you imagine that ANNE was when she realized that she had made the right choice?
• BERIT did not follow her intuition. She based the ordering of goods on analyses of previous sales, and succeeded. How satisfied (on a scale of 1 to 7) do you imagine that BERIT was when she realized that she had made the right choice?

In the same group (A), the “Employment” scenario described two heads of a computer enterprise, Kari and Finn, who employed a new data manager according to, respectively, an intuitive (based on a job interview impression) or a non-intuitive approach (based on the applicant’s record). One was successful and the other not. Group (A) participants were questioned as follows:

• KARI did not emphasize the information in the applications. She followed her intuition after the job interviews, and failed. To what extent (on a scale of 1 to 7) do you imagine that KARI will feel regret when she realizes that she had made the wrong choice?
• FINN did not follow his intuition. He based his decision making on the information provided in the applications, and succeeded. How satisfied (on a scale of 1 to 7) do you imagine that FINN was when he realized that he had made the right choice?

#### Within-Subjects Comparisons

In the first choice in group (A) (*n* = 31), both the intuitive and the non-intuitive decision maker succeeded in the “Ordering of goods” scenario; in the first choice in group (B) (*n* = 34), they both failed in the “Ordering of goods” scenario; in the first choice in group (C) (*n* = 30), they both failed in the “Employment” scenario; and, in the first choice in group (D) (*n* = 28), the intuitive and the non-intuitive decision makers both succeeded in the “Employment” scenario. These within-subjects designs allowed participants to directly compare intuitive and non-intuitive choices having the same outcome.

#### Between-Subjects Comparisons

In the second choice in group (A), the intuitive decision maker failed in the “Employment” scenario, while the non-intuitive one succeeded. In the second choice in group (B), the non-intuitive decision maker failed in the “Employment” scenario while the intuitive one succeeded. For participants in group (C), the second choice scenario was “Ordering of goods,” and the intuitive decision maker failed while the non-intuitive one succeeded. In the second choice in group (D), the non-intuitive decision maker failed in the “Ordering of goods” scenario while the intuitive one succeeded.

For the second choice scenario, we analyzed the ratings of the participants in a between-subjects design. We compared the ratings of participants of two scenarios and two groups, one in which the hypothetical decision maker made a successful (unsuccessful) intuitive choice, and one in which he/she made a successful (unsuccessful) non-intuitive choice in the same scenario. For instance, we compared the ratings of the participants in the “Employment” scenario in groups (A) and (B), where the intuitive decision maker failed in group (A) and the non-intuitive decision maker failed in group (B).

After receiving information about the respective outcomes, participants were asked to rate, for each choice, how much they thought that the hypothetical decision makers in the scenarios would regret choices leading to negative outcomes, and how satisfied they imagined they would be with choices having positive outcomes. Participants were also asked to rate how much responsibility (guilt, credit) they expected the decision makers to feel in relation to each outcome. As a further check of the hypothesized association between intuitive decisions and personal involvement, we included in the present study ratings of *self-esteem* as an additional dependent variable. Finally, the participants were asked to indicate how certain they were that the decision maker, after failing or succeeding, would choose the same approach (intuitive or non-intuitive) *next time* in a similar choice situation.

The dependent variables in Study 2 were, accordingly, *satisfaction, regret, responsibility* (following imagined good or bad choice outcomes), the extent to which the outcome would affect (increase or decrease) the decision maker’s *self-esteem*, and the likelihood of the decision maker choosing the same approach *next time*. We used 7-points Likert-type scales for all the dependent variables, bar *next time*, for which we used a 100% certainty scale.

### Results

The mean ratings of intuitive versus non-intuitive choices are presented in **Tables 1** and **2**. As can be observed in the tables (first row in each scenario), the participants anticipated that a decision maker ending up with a positive outcome from an intuitive choice would be markedly more satisfied than a decision maker achieving the same positive outcome from a non-intuitive choice. Similarly, the tables (second row in each scenario) show that the participants anticipated that a decision maker obtaining a negative outcome from an intuitive choice would regret more their choice than a decision maker obtaining the same negative outcome as a result of choosing the non-intuitive alternative.

**Table 1:** Mean ratings of satisfaction (positive outcomes), regret (negative outcomes), responsibility, self-esteem, and “next time” in the “Ordering of goods” scenario, Study 2.

	Positive outcomes				Negative outcomes			
	Intuitive	Non-intuitive	*P*	Cohen’s *d*	Intuitive	Non-intuitive	*P*	Cohen’s *d*
*Within-subjects comparisons:*								
Satisfaction	6.80	4.85	<0.001	2.61	-	-		
Regret	-	-			5.91	4.43	<0.001	1.32
Responsibility	6.27	4.74	<0.001	1.32	6.20	3.74	<0.001	2.33
Self-esteem	6.39	4.35	<0.001	2.18	2.74	4.14	<0.001	-1.64
Next time	76.6	80.8	0.182	0.23	28.6	46.0	<0.001	-1.02
*Between-subjects comparisons:*								
Satisfaction	6.78	5.93	<0.001	1.08	-	-		
Regret	-	-			5.81	5.07	0.042	0.54
Responsibility	6.17	5.06	0.002	0.98	6.19	3.93	<0.001	1.87
Self-esteem	5.90	4.84	<0.001	1.01	3.00	4.76	0.014	-0.66
Next time	81.7	76.0	0.282	0.28	24.6	44.8	0.004	-0.81

*Within-subjects comparisons = *t*-tests (two-tailed) for paired samples. Between-subjects comparisons = *t*-tests (two-tailed) for independent samples.*

**Table 2:** Mean ratings of satisfaction (positive outcomes), regret (negative outcomes), responsibility, self-esteem, and “next time” in the “Hiring employee” scenario, Study 2.

	Positive outcomes				Negative outcomes			
	Intuitive	Non-intuitive	*p*	Cohen’s *d*	Intuitive	Non-intuitive	*p*	Cohen’s *d*
*Within-subjects comparisons:*								
Satisfaction	6.50	5.57	<0.001	1.03	-	-		
Regret	-	-			5.53	4.97	0.024	0.49
Responsibility	6.21	5.00	<0.001	1.20	5.73	3.97	<0.001	1.47
Self-esteem	6.00	4.71	<0.001	1.30	3.27	3.70	0.051	-0.45
Next time	80.3	72.8	0.143	0.40	35.9	45.5	0.106	-0.43
*Between-subjects comparisons:*								
Satisfaction	6.76	6.10	0.011	0.64	-	-		-
Regret	-	-			5.94	4.60	<0.001	0.97
Responsibility	5.97	5.39	0.008	0.67	6.61	4.53	<0.001	1.92
Self-esteem	6.14	4.96	<0.001	1.32	3.48	3.62	0.653	-0.11
Next time	77.8	80.8	0.395	-0.21	28.3	46.9	0.001	-0.92

*Within-subjects comparisons = *t*-tests (two-tailed) for paired samples. Between-subjects comparisons = *t*-tests (two-tailed) for independent samples.*

As in Study 1, the participants attributed more responsibility (credit) to a decision maker for a positive outcome obtained through an intuitive choice than for the same positive outcome following a non-intuitive choice. Similarly, negative outcomes following an intuitive choice generated more responsibility (blame) than the same outcomes following a non-intuitive choice (**Tables 1, 2**, third row). As can be observed in the tables, the patterns of results are similar in between-subjects and within-subjects comparisons. Most of the differences are significant at a 0.001 level in both designs.

**Tables 1** and **2** (fourth row) show that the participants in both scenarios anticipated that the decision maker’s self-esteem would be markedly more improved following a successful intuitive choice than after an equally successful non-intuitive choice. The participants also assumed that negative outcomes would be slightly more detrimental for the self-esteem of the intuitive decision maker than for the non-intuitive decision maker.

As **Tables 1** and **2** (fifth row) show, the participants anticipated that it was far less certain that the decision maker would choose the same kind of option (intuitive or non-intuitive) next time if they had failed after making an intuitive choice than if they had failed after making a non-intuitive choice. Regarding successes, no significant differences were found.

### Discussion

Both scenarios showed that choosing the intuitive option was expected to lead to markedly stronger emotions, more regret following negative outcomes, and, in particular, more satisfaction with positive outcomes. We also found that the participants anticipated that choosing the intuitive versus the non-intuitive option would lead to markedly stronger feelings of responsibility for both positive and negative outcomes. All these effects were significant with both designs in both scenarios, although somewhat stronger, as expected from previous studies (e.g., Charnes et al., 2012), for within-subjects than for between-subjects comparisons.

The findings from the scenario studies—in particular, the findings of Study 2 in which participants were predicting *other* decision makers’ feelings—clearly support our original hypothesis. However, these findings primarily tap into participants’ *beliefs* about their own and others’ emotional responses to hypothetical choices. We also wanted to explore our hypothesis with respect to real-life decisions that the participants themselves had made. We therefore performed two autobiographical studies (Study 3 and 4) in which the participants— psychology students in Study 3 and experienced engineers in Study 4—were asked to describe examples of choice conflicts between intuitive and non-intuitive options from their own lives.

## Study 3

### Method

In Study 3, the participants were undergraduate psychology students at the University of Oslo (*N* = 88; male = 13, female = 75; *M*_age_ = 26.49 years, *SD* = 5.83), who volunteered to take part in the study.

The students were asked to write down an instance of a choice conflict between an intuitive option and a non-intuitive alternative from their private lives, and to indicate which option they had chosen. They were then asked to rate (on 9-point Likert-type scales) how certain they had been of making the correct choice just after the choice was made, how negative/positive they now considered the outcome of the choice to be, to what extent they regretted their choice, how satisfied/dissatisfied they were with the outcome, how responsible they felt for the outcome of their choice (in retrospect), and how responsible they considered others to be.

### Results

The participants described a wide range of choices: choice of partner, choice of holiday destination, choice of university, etc. A non-significant majority of the participants (56.8%; *p* = 0.241, 2-tailed, binomial test) reported an intuitive choice; that is, a choice where they had (finally) decided in favor of the intuitively appealing option. The mean ratings of intuitive versus non-intuitive choices are presented in **Table 3**.

**Table 3:** Mean ratings (scale 1–9) of chosen option after a choice conflict between intuitive and non-intuitive options, Study 3—Students.

	Intuitive	Non-intuitive	*T*	*p*	Cohen’s *d*
Certainty	4.62	3.84	2.151	0.035	0.46
Positivity	5.60	4.95	1.941	0.056	0.41
Satisfaction	5.94	5.03	2.597	0.016	0.54
Regret	1.72	2.61	2.759	0.010	-0.59
Responsibility, own	6.58	6.34	1.294	0.223	0.27
Responsibility, others	2.16	2.76	1.770	0.080	-0.37

*Between-subjects comparisons = *t*-tests (two-tailed) for independent samples.*

When the students were asked how certain they had been in making the correct choice just after the choice was made, those who had chosen the intuitive option reported a significantly higher certainty than those who had chosen the non-intuitive alternative (**Table 3**, first row). **Table 3** further shows that those who had made an intuitive choice considered the outcome (i.e., the consequences) of the choice as more positive than those who had chosen the non-intuitive alternative, and they were in retrospect much more satisfied with their choice than those who reported a non-intuitive choice.

The students who had chosen the intuitive alternative reported somewhat higher responsibility for the choice than those who had chosen the non-intuitive (analytic) alternative. They also attributed less responsibility to others. However, none of these differences were significant.

### Discussion

Our finding that the students who had chosen the intuitive alternative reported that they were more certain that they had made the correct choice than those who had chosen the non-intuitive alternative is in accordance with Simmons and Nelson’s (2006) “intuitive bias hypothesis,” which posits that, “because intuitions are often held with high confidence, people will choose intuitive options more frequently than equally valid non-intuitive options” (p. 411). This hypothesis implies a tendency of people to be more certain about intuitive than non-intuitive, analytic choices.

Since those who had reported a non-intuitive choice in retrospect were less satisfied with their choice, they, not surprisingly, also regretted more their choice. We interpret the finding that the participants were markedly more satisfied with their intuitive choices as being due to a higher degree of personal involvement in intuitive than in non-intuitive choices. We also consider the tendency to attribute somewhat less responsibility to others after making an intuitive choice as being in support of this interpretation (see also the section “General Discussion” below).

In sum, the findings of this study are compatible with those from Study 2.

## Study 4

The choices the young psychology students came up with in Study 3 were mostly choices from their personal lives. In Study 4, we performed the same study on experienced engineers, professionals who, in their work, are associated with an analytical approach and hence might be expected to make fewer intuitive choices than psychology students.

### Method

The participants were employees in a Norwegian engineering company (*N* = 99; male = 86, female = 13; *M*_age_ = 46.33 years, *SD* = 9.8) who volunteered to answer a web-based questionnaire about work-related decisions. They were first asked to describe briefly a choice conflict between an intuitive option and a non-intuitive alternative from their own professional life, as follows:

“You have probably experienced having the choice between two alternatives where objective facts and analytic reasoning support one of the options and your intuition or gut feeling tell you to choose the other […] Please recall and describe one example of such a choice dilemma.”

They then were asked the same questions as the students in Study 3, starting with which option they had chosen. Subsequently, they were asked to rate (this time on 7-point Likert-type scales) how certain they had been about making the correct choice, how negative/positive they now considered the outcome of the choice, the extent to which they regretted their choice, how satisfied/dissatisfied they were with the outcome, how responsible they felt for the outcome of their choice, and how responsible they considered others to have been for the final outcome.

### Results

The engineers came up with a wide range of choices within their professional context: accept or decline a new position (as leader) in the company; employ a person of foreign origin or a native Norwegian; choose a more expensive, well-known subcontractor or a much cheaper newcomer, etc. A significant majority (61.6%, *p* = 0.027, 2-tailed, binomial) of the engineers who had experienced a conflict between an intuitive option and a non-intuitive option ended up choosing the intuitive option. Mean ratings of intuitive versus non-intuitive choices are presented in **Table 4**.

**Table 4:** Mean ratings (scale 1–7) of chosen option after a choice conflict between intuitive and non-intuitive options, Study 4—Engineers.

	Intuitive	Non-intuitive	*T*	*p*	Cohen’s *d*
Certainty	5.21	4.79	1.433	0.155	0.29
Positivity	5.38	4.45	2.745	0.014	0.54
Satisfaction	5.85	4.68	3.634	0.002	0.72
Regret	1.80	2.61	2.453	0.026	-0.49
Responsibility, own	5.82	5.55	0.969	0.814	0.20
Responsibility, others	3.31	4.29	2.954	0.004	-0.70

*Between-subjects comparisons = *t*-tests (two-tailed) for independent samples.*

In the two retrospective studies (Study 3 and Study 4) combined, a significant majority of the 187 participants (59.4%, *p* = 0.013, 2-tailed, binomial test) reported choices from their own lives for which they had chosen the intuitive option.

### Discussion

The ratings of the experienced engineers in Study 4 replicate the trends found within the students’ responses in Study 3. The intuitive option was chosen with somewhat higher certainty, and the outcomes (consequences) of intuitive choices were perceived to be markedly more positive than the outcomes of non-intuitive choices. In accordance with this last finding, those who reported an intuitive choice were in retrospect also much more satisfied with their choice than those who had chosen the non-intuitive alternative. Probably because the engineers who had reported a non-intuitive choice in retrospect were less satisfied with their choice, they also regretted it more.

Engineers who had chosen the intuitive alternative felt in retrospect that they had a slightly higher, but not significantly different, responsibility for the choice, compared to those who had chosen the non-intuitive alternative. However, they rated others’ responsibility to be considerably less. Overall, the findings from both autobiographical studies go in the same direction as the very clear findings from the scenario studies (Study 1 and Study 2). In all the studies, the participants show a tendency to report stronger emotions in relation to the outcomes of intuitive choices than in respect of the outcomes of non-intuitive choices—in particular, more satisfaction following positive outcomes.

However, not all differences were significant. The somewhat mixed findings from the retrospective studies may be due to the large variability of the types of choice reported. The participants in these studies distinguished for themselves between the intuitive and the non-intuitive alternative, and their distinctions did not always make sense to us. It seems that some of them may have misunderstood how we in the studies described a conflict between an intuitive and a non-intuitive option.

## General Discussion

In the scenario study in which the participants themselves were asked to make a choice between an intuitive option and a non-intuitive alternative (Study 1), we found that there was a significant tendency in the three concrete scenarios toward choosing the intuitive option. In the two retrospective studies a significant majority reported choices from their own lives for which they had chosen the intuitive option. Thus, the present studies’ results indicate a propensity of people to favor intuitive options in decision making.

In the scenario studies, the intuitive option was found to lead to stronger emotions—in particular, to more satisfaction in the case of a positive outcome. Furthermore, results from the retrospective studies point us toward the same inference. In fact, the findings from all four studies indicate that intuitive choices are associated with intensified emotional experiences—but why might this be so?

### Decision Makers Feel More Personally Involved in Intuitive Choices

Our findings clearly suggest that intuitive choices give a stronger feeling of responsibility for the outcome than non-intuitive choices. Decision makers who end up with a positive or a negative outcome after choosing the intuitive option are, in the studies, consistently anticipated to experience stronger feelings of responsibility (credit or guilt) than decision makers who obtain the same outcome after choosing the non-intuitive alternative.

The relationship between responsibility and agency has long been recognized. The ancient Greek philosopher Epicurus (341–270 BC) postulated more than 2,000 years ago that we acquire the idea that we are causal agents through observing that human beings, including ourselves, are praised and blamed for their actions (e.g., Frith, 2013). More recently, the relationship between agency and responsibility has been explored experimentally (e.g., Woolfolk et al., 2006; Nordbye and Teigen, 2014). For example, Woolfolk et al. (2006) found that, the more an actor is identified with an action, the more appropriate people find it that responsibility should be assigned to that actor.

In our studies, we did not ask the participants explicitly to assess a decision maker’s degree of personal involvement or experienced self-agency. However, the differences in responsibility assessments indicate that, consistent with Epicurus’ past insight, intuitive decision makers are believed to be more personally involved in their decisions than non-intuitive decision makers.

In Study 2, the participants anticipated that, in both scenarios, decision makers who were successful after choosing the intuitive option would experience markedly increased self-esteem compared to those who were successful after choosing the non-intuitive alternative. Similarly, the participants anticipated that decision makers who ended up with negative outcomes after choosing the intuitive option would experience less self-esteem than those who ended up with the same negative outcome after choosing the non-intuitive alternative. These differences in predicted self-esteem also suggest that decision makers are considered to be more personally involved in their intuitive than in their non-intuitive choices.

In the two autobiographical studies (Study 3 and Study 4), the participants judged in retrospect the outcomes of their intuitive choices as more positive than the outcomes of their non-intuitive ones, suggesting that they were personally more involved in their intuitive than in their non-intuitive choices.

Altogether, the above findings indicate that intuitive choices increase the feeling of personal involvement more than non-intuitive choices do.

### Personal Involvement Mediates Increased Emotional Experience

Inferring that choosing an intuitive option seems to intensify the (anticipated) emotions associated with the choice outcomes, as well as increase the decision maker’s personal involvement, we might then consider whether the stronger personal involvement gives rise to stronger emotional experiences, or vice versa? According to Chambon and Haggard’s (2012) findings, it seems reasonable to assume that a strengthened experience of personal involvement is a consequence of selecting the intuitive option. This experience thus precedes the emotional reactions in the choice outcomes. So, the increased personal involvement following an intuitive versus a non-intuitive choice may well mediate the stronger emotions in the choice outcomes of intuitive choices. Of course, the effect may go the other way as well, and stronger emotions *could*, in turn, contribute to a further increase in the feeling of personal involvement in intuitive versus non-intuitive choices.

### Intensified Positive Emotions Reinforce Intuition Bias

The present studies clearly support our hypothesis that people are more emotionally affected by the outcomes of intuitive choices than by those of non-intuitive choices. However, do these intensified feelings following intuitive choices also contribute to people’s exaggerated tendency to go with their gut, reinforcing intuition bias?

We noted above that one conventional explanation for intuition bias focuses on the nature of people’s expected *negative* counterfactual thoughts if their decisions were to turn out badly (e.g., Risen and Gilovich, 2008). However, the present data indicate that intuitive choices intensify anticipated positive emotions after successful outcomes more than anticipated negative emotions after failures. This anticipation is in accordance with studies showing that increased fluency, in fact, triggers *positive* affect in the choice outcomes (e.g., Reber et al., 2004; Topolinski and Strack, 2009).

We know from prior research that people expect their choices to have positive rather than negative outcomes (e.g., Bracha and Brown, 2012). In fact, that is why people make the choices they do. If they had believed a choice would turn out badly, they would have selected a different option. So, since the increment from positive outcomes is greater than the decrement for negative outcomes, and positive outcomes are more commonly expected, anticipated positive emotions should play a more important role for decision making than anticipated negative emotions.

It has been affirmed that “the experience of self-agency is fundamental to human self-perception” (Aarts et al., 2009, p. 967). An increased feeling of self-agency and personal involvement following intuitive choices should then, in themselves, reinforce people’s tendency to go with their gut, on top of the anticipation of intensified emotions in respect of the commonly expected positive outcomes.

Accordingly, we find it plausible that the increased feeling of personal involvement following intuitive choices and the intensified emotions associated with the positive outcomes of such choices contribute to people’s tendency to prefer intuitively appealing over equally valid non-intuitive options.

However, we also found (in Study 2) that intuitive decision makers who were *informed* that their intuitive choice had led to a negative outcome, were to a lesser extent expected to choose the same kind of option next time, compared to those who were informed that their choice of a non-intuitive option had led to a negative outcome. This finding indicates that people are more willing to learn from their failed intuitive choices than from their failed non-intuitive choices, which in turn may moderate somewhat the tendency to go with one’s gut in decision making.

### Limitations and Further Studies

A limitation with scenario studies like those reported here is that how one believes that oneself (Study 1) and others (Study 2) will feel, can be different from how oneself and others will, in fact, react and feel in real situations. This limitation was partly the motivation for the retrospective studies, where we asked the participants to come up with choice conflicts they themselves had experienced. However, even retrospective studies can only measure thoughts and feelings viewed from a distance. This limitation is particularly obvious for the study of emotions (e.g., Frijda and Zeelenberg, 2001). Our studies should therefore be followed up by studies of real life decision making.

One particular weakness with our studies is that we did not measure the duration of the decision process for “intuitive” versus “non-intuitive” options. We assumed (and explained to the participants) that intuitive choices are immediate, and decision latencies accordingly much shorter than those determined by an analytic process. In contrast to scenario and retrospective studies, one can in studies of real life decision making easily measure reaction times and in that way control for this conjecture. We suggest that in particular one finding should be examined further in real life decision making situations, namely responsibility and personal involvement, which both in Study 1 and 2 were rated higher for intuitive choices. This finding was supported by the findings from the retrospective studies. It may, however, be objected that time and effort spent on a real life decision task might also increase involvement and responsibility and thus reduce the differences we found between deliberative and intuitive choices.

## Conclusion

Several explanations, well supported by empirical evidence, have been advanced regarding the exaggerated tendency of individuals to follow their intuitions. Our findings indicate an additional potential reason for this tendency, based on the different experiences associated with the outcomes of intuitive and non-intuitive choices. People seem to be more personally involved in their intuitive choices, and experience more intensified emotions—particularly in relation to the positive outcomes of such choices. We conclude that the increased feeling of personal involvement following intuitive choices, and the markedly intensified emotions, actual and anticipated, in the positive outcomes of them, constitute another, so far overlooked, reason for intuition bias.

## Ethics Statement

The research project was approved by Department of Psychology’s (University of Oslo) Research Ethics Committee (ref number 1088630). All the questionnaires started with an informed consent form that the participants had to tick off before proceeding to the questionnaire. No person-identifiable information was recorded.

## Author Contributions

GK and GN worked together on all parts of the study. GK as main author conceived the idea behind the study and performed the initial literature research.

## Conflict of Interest Statement

The authors declare that the research was conducted in the absence of any commercial or financial relationships that could be construed as a potential conflict of interest.
